# A Quantitative Study on Crucial Food Supplies after the 2011 Tohoku Earthquake Based on Time Series Analysis

**DOI:** 10.3390/ijerph17197162

**Published:** 2020-09-30

**Authors:** Xiaoxin Zhu, Yanyan Wang, David Regan, Baiqing Sun

**Affiliations:** 1 School of Business, Qingdao University of Technology, Qingdao 266525, China; zhuxx0813@hotmail.com; 2 School of Public Policy and Management, Tsinghua University, Beijing 100084, China; 3 School of Foreign Studies, China University of Petroleum, Qingdao 266580, China; Q20200003@upc.edu.cn; 4 School of Management, Harbin Institute of Technology, Harbin 150001, China; baiqingsun@hit.edu.cn

**Keywords:** natural disaster, emergency response, food supplies, time series analysis, Tohoku earthquake

## Abstract

Awareness of the requested quantity and characteristics of emergency supplies is crucial for facilitating an efficient relief operation. With the aim of focusing on the quantitative study of immediate food supplies, this article estimates the numerical autoregressive integrative moving average (ARIMA) model based on the actual data of 14 key commodities in the Sendai City of Japan during the 2011 Tohoku earthquake. Although the temporal patterns of key food commodity groups are qualitatively similar, the results show that they follow different ARIMA processes, with different autoregressive moving averages and difference order patterns. A key finding is that 3 of the 14 items are significantly related to the number of temporary residents in shelters, revealing that the relatively low number of different items makes it easier to deploy these key supplies or develop regional purchase agreements so as to promptly obtain them from distributors.

## 1. Introduction

Emergency events, such as Hurricane Katrina in the US in 2005, the 2011 Tohoku earthquake in Japan and the 2019 novel coronavirus (COVID-19) outbreak in China, can strike a community with little warning and leave much devastation and many casualties behind. Public officials are faced with further critical questions after a disaster occurs. The most heavily criticized aspect of official responses is often one of inefficient relief effort that does not punctually deliver the critical supplies needed to disaster-hit areas. How to respond to such emergencies in the most efficient manner in order to minimize the loss of life and maximize the efficiency of the rescue operations is the ultimate aim of disaster relief [1,2,3,4].

To effectively combat disasters, it is essential that emergency managers are able to predict and analyze potential dangers and develop the necessary strategies for mitigating adverse conditions and responding to them in an effective manner [5]. The main goal of emergency response efforts is to provide assistance to disaster victims as soon as possible [6]. An area that is in need of research is the estimation of immediate resource requirements, i.e., those supplies that satisfy needs generated by both the disaster itself and the ensuring response. For an extended period of time, this has been identified by FEMA as a high priority research topic [7,8].

Japan is a country that is prone to natural disasters, especially earthquakes. The 2011 Tohoku earthquake caused a major leakage in a regional nuclear power plant, prolonging the evacuation and bringing new challenges to supplies management. The Tohoku experience demonstrated that a lack of an efficient response based on an understanding of the characteristics of supplies is bound to have negative consequences with respect to how these supplies affect the lives of individuals. There were failures in logistical planning, with untimely and lagged delivery of requested goods. For example, some affected residents did not receive any food supplies for one week due to a shortage of rescue personnel and transportation vehicles. Furthermore, even though the supplies that victims need may change over time, the amount of supplies were planned in a fixed manner without concern for the actual concrete situation. Finally, there were deliveries of supplies that were of a low priority: some less essential food supplies, such as perishables like fruit and vegetables, were delivered at the wrong time; some letters and books that had a lower priority arrived during the initial stage of the disaster when they were less needed, taking up much needed storage space and even causing severe congestion. Thus, a key objective in emergency logistics is to establish a functional framework for efficient relief operations by estimating the timing of what is demanded, the quantity of the relief goods demanded, and what the crucial commodities are.

In order to achieve the goal described above, the supply of emergency materials, such as food, clothing and medical supplies, is essential, with the emergency response planning of supply deployment and allocation being determined in such a way that they are readily available when needed. Nonetheless, allocating such disaster response facilities also requires consideration of the stochastic nature of the problem as it pertains to the prediction of the demand of supplies and different phases in the disaster lifecycle, such as preparedness and response. Therefore, the need arises for a modeling approach that is able to provide decision makers with early estimates of the future quantity of supplies based on historical time series data. The goal, then, is to predict the trends in emergency supplies management so as to facilitate early public health responses to increase the efficiency of rescue operations and minimize mortality rates.

To address these issues, quantitative research, involving 14 affected food supplies in Sendai City in Japan during the 2011 Tohoku Earthquake, is employed. The remainder of the paper is organized as follows. Section 2 begins with an overview of relevant literature and debates surrounding emergency supplies and a framework for understanding overall emergency response processes. Section 3 presents the region of study, data gathering and research methodology. Section 4 presents the results of time series analyses (ARIMA and LSM) aimed at exploring crucial supplies and temporal patterns. Section 5 and Section 6, respectively, discuss the results and conclude with a summary of our key findings.

## 2. Literature Review

### 2.1. Emergency Supplies Management

The objective of disaster response in the emergency relief chain involves deploying and dispatching commodities and resources (e.g., medical materials and personnel, specialized rescue equipment and rescue teams, food, etc.) to distribution centers in affected areas as soon as possible so that relief operations may be accelerated [9,10]. Some research efforts have been devoted to the analysis of the management of emergency materials. These are introduced in turn.

#### 2.1.1. What Is Demanded in Terms of Timing, Type and Size

Rivera-Royeroa, et al. [11] put forward a dynamic methodology for distributive decision-making processes. The model allows for different levels of dynamic demand priorities among the affected population. Put another way, it is likely that some groups require relief items with greater urgency than others. Some of the factors that may cause differences in priority levels are to do with different elapsed times since the occurrence of the disaster and the type of item being requested. Holguín-Veras and Jalle [7] develop numerical estimates of the requirements that were needed during Hurricane Katrina and their temporal patterns through an analysis of the data provided by the Federal Emergency Management Agency (FEMA). Zhao, et al. [12] consider both the post-disaster condition of buildings and the critical factor of human choice that affects evacuees’ decisions, proposing a forecasting method to estimate the time-varying demand for shelters.

#### 2.1.2. Quantitative Estimation of Supplies in Urgent Situations after Disasters Hit

Kemball-Cook and Stephenson [13] investigate the refugee relief operations in Somalia in 1981 through a description of how over 99% of food received at Mogadishu succeeded in going on to reach affected areas, with detailed attempts to diagnose the contributing factors. Haghani and Oh (1996) propose two heuristic algorithms dealing with multiple commodities and multiple modes of transportation based on the concept of a time-space network [2]. Barbarosoglu and Arda [14] propose a two-stage stochastic programming model to plan the transportation of vital first-aid commodities to disaster-affected areas during emergency responses. Focusing on rapid needs assessment operations conducted immediately after a disaster, Balcik and Yanıkoğlu [15] identify the urgent needs of affected community groups, addressing the problem of selecting the sites to be visited by the assessment teams during a fixed assessment period and constructing assessment routes under travel time uncertainty.

#### 2.1.3. Emergency Response of Supply Requests Based on Concrete Situations

Considering the existence of different priority levels among emergency supplies, Son, et al. [16] develop a model based on a repertoire of resilience strategies that manipulate essential resources, thereby facilitating performance adjustment. Rodríguez-Espíndola, et al. [17] develop a system for emergency preparedness that enables the identification of required organizations under the circumstances of the respective emergency, providing tailored operations that prevent material and human convergence and shortages. Ozdamar, Ekinci and Kücükyazici [10] develop a model that regenerates plans incorporating new requests for aid materials, new supplies and transportation means that become available during the current planning time horizon.

### 2.2. Response Processes of Supply Management in Sendai City in the 2011 Tohoku Earthquake

**Figure 1:**
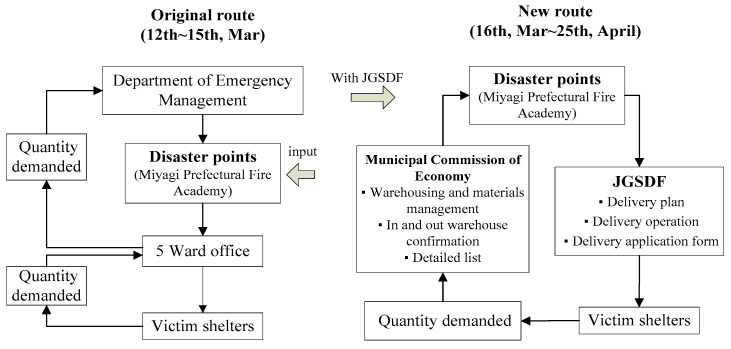
The scheduling process in Sendai City during the 2011 Tohoku earthquake.

Managing disaster response operations is a challenging task that requires the consideration of many stakeholders under conditions of time pressure, risk and uncertainty [18]. As shown in Figure 1, from 12 to 15 March, after the occurrence of the Tohoku Earthquake, Sendai City delivered food supplies to disaster points in Miyagi Prefectural Fire Academy, as ordered by the Department of Emergency Management. From there, the supplies were transported from the disaster points to 5 Ward Offices and then on to victim shelters. After the Japan Ground Self-defense Force (JGSDF) joined the operation, the scheduling route changed. Orders were now made by the Municipal Commission of the Economy, with supplies still delivered to the disaster points as in the previous process. However, from here, it was now the JGSDF who devised the delivery plans to get the necessary supplies to the shelters.

## 3. Models and Method

### 3.1. Region of Study

On 11 March 2011, Japan experienced the strongest earthquake in its recorded history. The quake had a magnitude of M = 9.1, making it one of the most devastating earthquakes to ever hit Japan. It struck below the North Pacific Ocean, 130 km (81 miles) east of Sendai, the largest city in the Tohoku region in the northern part of Honshu island [19]. As of June 10, 2016, according to the official data, the number of confirmed deaths stands at 15,894.

**Figure 2:**
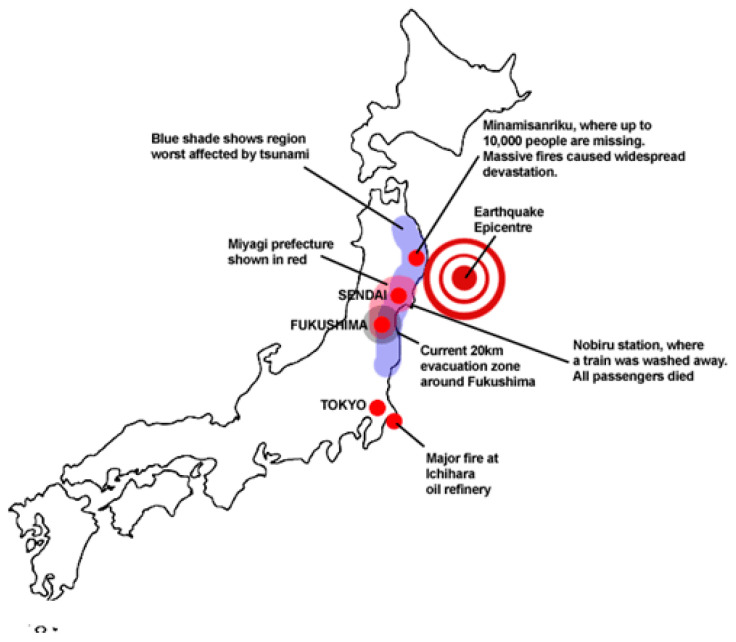
Map of Tohoku earthquake zones (source: https://100gf.files.wordpress.com/2011/03/japanmap.gif).

Less than an hour after the earthquake, the first of many tsunami waves hit Japan’s coastline. These waves reached run-up heights (how far the wave surges inland above sea level) of up to 128 feet (39 m) at Miyako city and traveled inland as far as 6 miles (10 km) to Sendai. It was along the Sanriku Coast, which runs from around 50 to 200 km north of Sendai, that the narrow bays focused the tsunami waves, generating the largest inundation heights and run-ups [20]. An estimated area of approximately 217 square miles (561 square kilometers) was left flooded. The map in Figure 2 shows the major areas of damage in Japan, including the Fukushima nuclear plant, the exclusion zone around the plant, and the locations of Sendai and Minamisanriku, two of the cities that were badly damaged in the disaster [21]. Due to the scale of the devastation in this disaster, Sendai City was chosen to be a case study, being that it is representative of a city responding to real time needs in a disaster context.

### 3.2. Data Gathering

**Figure 3:**
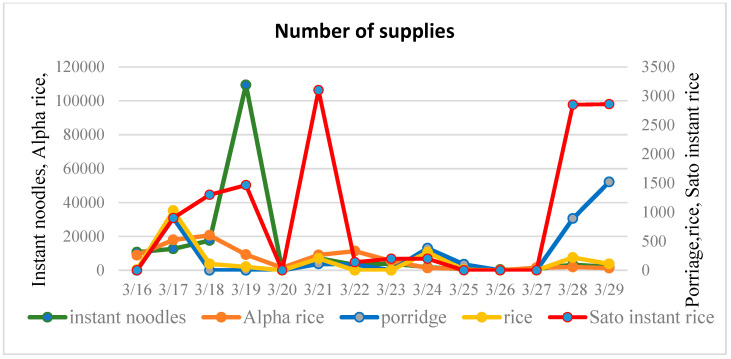
Supply of rice and noodle products.

**Figure 4:**
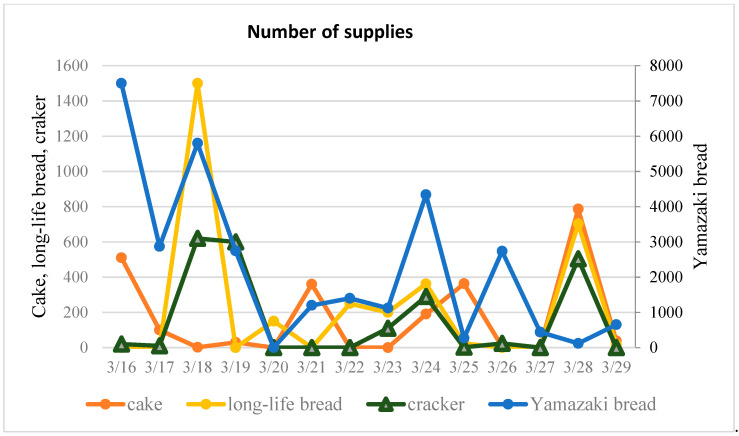
Supply of wheat-based commodities.

**Figure 5:**
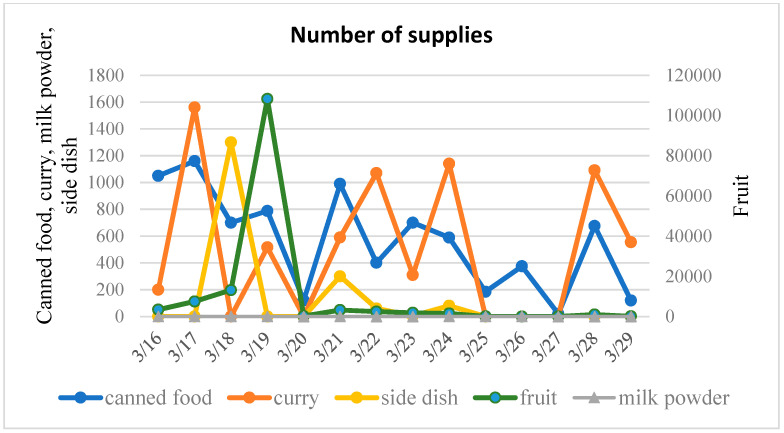
Supply of other goods.

Data from the Bureau of Economy, Trade and Industry from the Sendai Department of Industrial Policy was collected and analyzed [22]. In order to analyze the characteristics of different types of supplies, 14 food commodities that were supplied during the disaster are categorized into three groups: rice and noodle commodities, wheat-based products and other goods (see Figure 3, Figure 4 and Figure 5).

**Figure 6:**
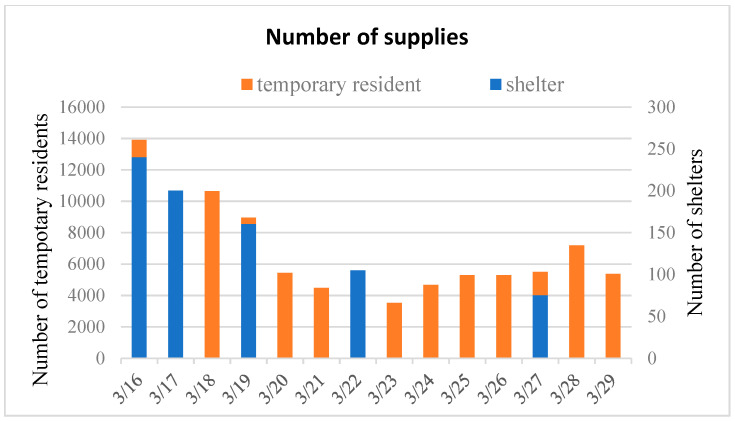
Number of temporary shelters and residents.

The number of temporary shelters and their respective residents from 16 to 29 March is shown in Figure 6. In addition to forecasting quantitative demand, the relationship between the residents in temporary shelters and the quantity of food supplies is analyzed in order to explore key commodities.

### 3.3. Time Series Analysis

**Figure 7:**
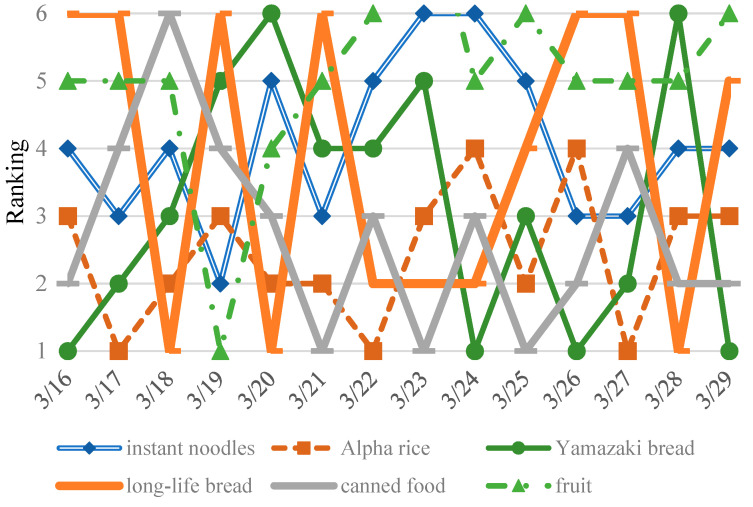
Temporal distribution of primary supplies over 14 days.

In emergency management, the accurate forecasting of dynamic temporal patterns of requested resources is indispensable. Time-series approaches, such as LSM (the Method of Least Squares), ARIMA (Autoregressive Integrated Moving Average Model), SES (Simple Exponential Smoothing Model) and HWES (Holt–Winters Exponential Smoothing Model), have been applied as an effective non-explanatory mean to predict future trends based on historical data. Time series are relatively simple to establish and require less detailed information, meaning that they are a reasonable method for forecasting the required demand of supplies. A high performing time-series model will facilitate the understanding required for decision making so as to make early response more efficient and better coordinated [23]. An initial exploration of the application of this method is employed by estimating the temporal request percentage for instant noodles and Alpha rice, Yamazaki bread and long-life bread, and canned food and fruit from the respective groups of rice and noodle products, wheat-based commodities and other goods. Figure 7 shows the relative ranking of these representative food relief goods, calculated by the actual percentage each day takes up for the total of each commodity. The relative ranking goes from one to six, with the request priority ranking from high to low. It can be seen that, despite similar variation in food supplies, their respective priority of requests changes over time. Therefore, it is necessary to further explore the trend of different commodities.

#### 3.3.1. ARIMA

The ARIMA modeling procedure is an established method and is extensively applied in forecasting time series [24]. An ARIMA process is characterized by three parameters, p, d and q, where p denotes the number of autoregressive terms, d the number of times the series needs to be differentiated before it becomes stationary and q the number of moving average terms. For this model type, the transformation of the original values might be necessary in coping with non-stationary series, in which at least one order of differencing is used in order to obtain a stationary series [25]. A model for an ARIMA process is a combination of an autoregressive (AR) process model and a moving average (MA) process model of the i^th^ integration in the series. In general, an autoregressive (AR) process can be modeled as [7]

(1) $$\left(Y_{t}-\delta_{t}\right)=\alpha_{1}\left(Y_{t-1}-\delta_{t-1}\right)+\alpha_{2}\left(Y_{t-2}-\delta_{t-2}\right)+\cdots +\alpha_{p}\left(Y_{t-p}-\delta_{t-p}\right)+u_{t}$$

where $Y_{t}$ is a $p^{th}$-order autoregressive or AR(p) process, δ is its mean, and $u_{t}$ is an autocorrelated random error term with zero mean and a constant variance $\sigma^{2}$ (i.e., white noise). In the same way, a moving average (MA) process, one that is simply a linear combination of white noise error terms, can be modeled as

(2) $$Y_{t}=\mu +\beta_{0}u_{t}+\beta_{1}u_{t-1}+\beta_{2}u_{t-2}+\cdots +\beta_{q}u_{t-q}$$

where $Y_{t}$ is a $q^{th}$-order moving average or MA(q) process, μ constant and u, as before, is the white noise stochastic error term. Working from the assumption that it is stationary, when a process has characteristics of both AR and MA it is therefore an autoregressive and moving average (ARMA) process and can thus be modeled as

(3) $$Y_{t}=\theta +\alpha_{1}Y_{t-1}+\alpha_{2}Y_{t-2}+\cdots +\alpha_{p}Y_{t-p}+\beta_{0}u_{t}+\beta_{1}u_{t-1}+\cdots +\beta_{q}u_{t-q}$$

where θ is a constant, and there are *p* autoregressive and *q* moving average terms. An autoregressive moving average (ARMA) model can be extended to autoregressive integrated moving average (ARIMA) models through a transformation and/or differencing procedure applied to the original values in order to obtain a stationary process.

#### 3.3.2. The Method of Least Squares

The efficient operation of emergency response systems requires the accurate forecast of the demand for supplies and of the expected load in the next period of the response. The forecasting methods that have been most used can be broadly categorized as classical time series and regression methods, artificial and computational intelligence methods, and hybrid approaches [26]. In more recent years, as the most widely used method for determining the position of the trend line of a given time series, the least square method (LSM) has been commonly used to find or estimate the numerical values of parameters so as to fit a function to a set of data and characterize the statistical properties of estimates.

The repeated measurements $y_{j}$ can be treated as the sum of the (unknown) quantity x and the measurement error $\varepsilon_{j}$,

(4) $$y_{j}=x+\varepsilon_{j}$$

The quantity x should be determined such that the sum of squares of the errors $\varepsilon_{j}$ is a minimum,

(5) $$\sum_{j}\varepsilon_{j}^{2}=\sum_{j}{\left(x-y_{j}\right)}^{2}=min$$

The method of least squares can also be used in cases where the measured quantities $y_{j}$ are not directly related to the unknown x, but rather indirectly, i.e., as a linear (or also nonlinear) combination of several unknowns XI, X2, … [27]. Because of the great practical significance of this method, in order to explore the relationship between the number of residents in temporary shelters and food supplies, in our model, the first step is to use an ARIMA model to forecast the time series and the quantity of food supplies. Due to the trend line that LSM determines technically being one of a best fit, LSM is used to establish a mathematical relationship between the time factor and the given variable. The professional software Eviews 10.0 is performed to conduct the analysis.

## 4. Results

### 4.1. ARIMA Model of Food Supplies

**Figure 8:**
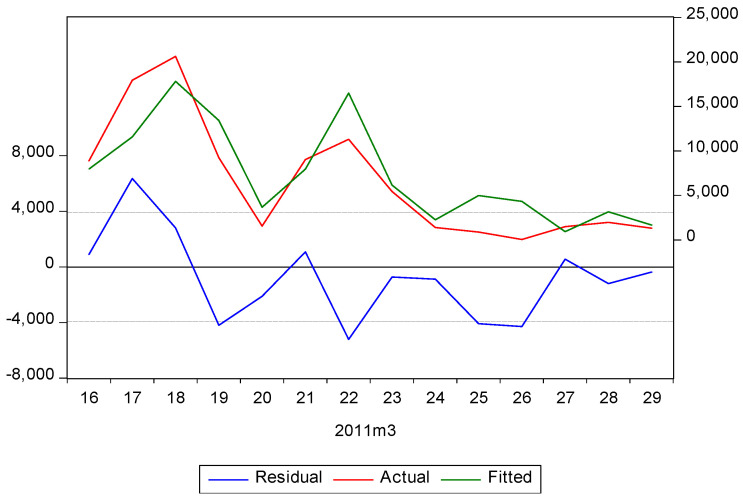
The fit figure of Alpha rice estimated by ARIMA.

Taking Alpha rice as an example in order to estimate the time trend of food supplies in the Tohoku earthquake, Table 1 shows the statistics of an ARIMA model computed by the outer-product-of-gradients (OPG). The R-squared value shows that an approximate 75.9% of the variance can be explained by the ARIMA model for Alpha rice. The probability (*F*-statistic) = 0.007 < 0.01 suggests that the model fit is adequate at a significance level of 10% (*p* < 0.01). Figure 8 presents the fitting curve of Alpha rice estimated by ARIMA, where it can be seen that the change pattern is similar.

Table 2 shows the ARIMA model estimation of 12 types of food supplies with statistics of different structures. Apart from crackers and milk powder, where no model was found, the majority of the models have the same AR structure, albeit with different parameters and difference order patterns.

### 4.2. The Relation between Residents and Food Supplies

As mentioned above in Section 3, the least squares method (LSM) is applied so as to estimate the regression coefficient [27]. Therefore, LSM (NLS and ARMA) is used here to explore the relations between the number of temporary shelters and different types of food supplies. As shown in Table 3, there is an item in each group which is significantly related to the variation in the number of residents: Alpha rice, Yamazaki bread and canned food. Detailed correlation analysis—carried out below—needs to be performed for further understanding so as to accurately interpret the results.

As can be seen in Table 4, Table 5 and Table 6, the respective T-statistic tests show that the number of residents is significantly related to the supplies of Alpha rice, Yamazaki bread and canned food. The R-squared value means that an approximate 28%, 27% and 57% of the variance can be explained by the model for Alpha rice, Yamazaki bread and canned food, respectively. It is possible that there may be other predictor (explanatory) variables that are not included in the current model. For Alpha rice (see Table 3), the *F* value of 4.46 > 3.285 suggests that the model fit is adequate (at 12 degrees of freedom). The *F*-test in the Yamazaki bread model (see Table 4), *F* value = 4.622 > 3.285, suggests that the model fit is adequate (at 12 degrees of freedom) at a significance level of 10% (*p* < 0.01). In the canned food *F*-test, an *F* value of 15.925 > 10.044 suggests that the model fit is adequate at a significance level of 1% (*p* < 0.01).

## 5. Discussion

From the literature review and based on statistical analysis of food supplies in the Tohoku earthquake, estimates are given of the partial relative ranking of request priorities, ARIMA models and the relationship between the number of residents and supply consumption. Such results were indicative of the feasibility of the established framework and provide ideas for the future exploration of the emergency supplies needed in disasters.

### 5.1. Temporal Evolution of Supplies Demanded in the Tohoku Earthquake

The temporal evolution of emergency supplies is complex, and in it, the request priority at different times can vary greatly. On 16 March, the request priority ranking is Yamazaki bread, canned food, instant noodles, fruit and long-time bread. As the time period progresses, the ranking constantly changes. In addition, the analysis also indicates that during the 14 days from 16 to 29 March, although the ranking varies in a dynamic fashion, Yamazaki bread, Alpha rice, canned food, long-life bread and fruit each had respective requests of 4 times, 3 times, 3 times, 3 times and once. This could indicate that some specific commodities are needed frequently, with each having a high priority.

The analysis revealed that it is essential to explore the request features of emergency supplies based on the concrete response situation, consistent with the findings of previous studies that emphasize the importance of requirement forecasting for response efficiency [7,28]. The potential benefits of being aware of the request ranking of emergency supplies are that it may aid decision makers and the early preparations for inventory management and material collection. Through this, insufficient inventory or the untimely collection of emergency supplies, as happened in the Tohoku earthquake, might be avoided to a certain extent.

### 5.2. Estimated Time Series of Food Supplies

The demand forecasting for the time series of commodities after natural disasters is especially important in emergency management [29,30]. The results show that, though the temporal patterns of food supplies are qualitatively similar, they follow different ARIMA processes. The majority of the ARIMA models for the 14 food supplies have the same structure, albeit with different parameters; that is, 12 food supplies tend to have a different number of autoregressive items and difference order patterns without following the integrated moving average process. In addition, no ARIMA model available was found for milk powder and crackers. This is most probably due to the non-linearity and non-stationarity of the data for these two commodities, thus not suiting the ARIMA model, and the lack of resources, their subsidiarity or irregular supply, which occasionally occurs in response practice.

The statistical estimation of the time-series (ARIMA) model results clearly reveal that the forecasting demand and time series of resource requirements could be modeled by using statistical tools. The logistical estimation of supplies demanded to provide relief to disaster victims and the appropriate planning management of these resources are critical to reduce any suffering caused [17]. However, since the time series of some supplies demanded in disasters is nonlinear, the integration of an ARIMA model and other empirical models in order to increase the forecasting accuracy provides insights into the future direction of emergency supply forecasting. The distribution of demand seen in the model built in this case study indicates a desirable direction for further research that aims to predict the trends for supplying relief goods.

### 5.3. Crucial Commodities of Food Supplies

Previous studies on demand calculation mainly focus on demand forecasting methodology, with many neglecting the checklist of critical supplies and practical background [4]. Models for the distribution of relief supplies often assume their immediate availability upon the occurrence of a natural disaster. However, such an assumption is not always applicable in concrete settings [11]. A key finding is that the temporal distribution of supplies shows the relative importance of different commodities as a disaster unfolds. Three of the 14 food supplies—Alpha rice and canned food at a significance level of 0.1 and Yamazaki bread at a significance level of 0.01—are significantly related to the number of evacuees in shelters. This is possibly due to regional preferences in taste, properties of the commodities that give them particular advantages and convenience, along with satiety duration and intensity. This reveals that a relatively low number of different items may enable the deployment of these key supplies with relative ease or the development of regional purchase agreements in order to promptly obtain them from distributors.

## 6. Conclusions

This study aims to develop a conception and framework to estimate demanded supplies and explore the characteristics of crucial supplies so as to facilitate early preparations and the efficient deployment of emergency supplies. Taking the 2011 Tohoku earthquake as a case study, statistical models show promise in representing how time series analysis can deal with emergency supplies and shed light on exploring the potential principle of supplies in the initial stage of a disaster. Furthermore, the analytical results of crucial commodities show that an exploration of the characteristics of supplies matters when making strategic pre-disaster decisions, especially concerning the collection of supplies and the future deployment of inventory. In addition, two general directions for future research can be considered. One is to explore the temporal pattern of other emergency supplies, for example medical supplies and clothing, by expanding the ARIMA models integrated with other empirical methods properly in a wider range of emergency events, since they may provide estimates of future needs. The other concerns the possibility that the relation of more characteristics of temporary residents in shelters could be explored so as to study the crucial supplies for disaster relief operations. It is our hope, then, that the findings of this paper may be used in the future, either by ourselves or by other researchers, to explore these further avenues.

## Figures and Tables

**Table 1:** Coefficient covariance computed using outer-product-of-gradients (OPG) of Alpha rice

Variable	Coefficient	Std. Error	*t*-Statistic	Prob.
C	6968.511	7320.139	0.952	0.366
AR (1)	1.184	0.205	5.763	0.000
AR (2)	−1.009	0.229	−4.401	0.002
AR (3)	0.710	0.242	2.931	0.017
SIGMASQ	9,831,953	4,665,112	2.108	0.064
R-squared	0.759	Mean dependent var		6515.643
S.E. of regression	3910.773	Akaike info criterion		19.870
*F*-statistic	7.075	Schwarz criterion		20.098
Prob (*F*-statistic)	0.007	Durbin–Watson stat		1.476

**Table 2:** Analysis of variance (ANOVA).

	Item	ARIMA Model	Parameters			
			Type	Coefficient	*t*-Statistic	Sig.
1	Instant noodles	(2,2,0)	AR (1)	−1.034	−3.594	0.007
			AR (2)	−0.552	−1.970	0.084
2	Rice	(3,1,0)	AR (1)	−1.008	−2.734	0.026
			AR (2)	−0.844	−2.062	0.073
			AR (3)	−0.589	−2.146	0.064
3	Alpha rice	(3,2,0)	AR (1)	1.184	5.763	0.000
			AR (2)	−1.009	−0.402	0.002
			AR (3)	0.710	2.931	0.017
4	Porridge	(1,2,0)	AR (1)	−0.778	−4.027	0.003
5	Sato instant rice	(1,2,0)	AR (1)	−0.729	−3.046	0.014
6	Yamazaki bread	(1,1,0)	AR (1)	−0.672	−3.005	0.013
7	Long-life bread	(3,1,0)	AR (1)	−0.926	−2.668	0.028
			AR (2)	−0.836	−2.251	0.055
			AR (3)	−0.681	−2.797	0.023
8	Cake	(2,2,0)	AR (1)	−0.988	−2.783	0.024
			AR (2)	−0.758	−1.919	0.091
9	Canned food	(1,1,0)	AR (1)	−0.779	−3.810	0.003
10	Curry	(1,1,0)	AR (1)	−0.711	−3.304	0.008
11	Side dish	(2,1,0)	AR (1)	−0.920	−4.817	0.001
			AR (2)	−0.870	−7.246	0.000
12	Fruit	(3,1,0)	AR (1)	−0.802	−2.586	0.032
			AR (2)	−0.665	−1.935	0.089
			AR (3)	−0.566	−2.114	0.067

**Table 3:** Correlation coefficient results.

	Category		Item	Coeff.	SE	*t*-Statistic	Prob.
Number of residents	I Rice and noodle products	1	instant noodles	3.4100	2.6454	1.2891	0.2217
		2	rice	−0.0045	0.1189	−0.0375	0.9707
		3	Alpha rice	1.2037	0.5599	2.1498	0.0527 *
		4	porridge	−0.0094	0.0473	−0.1997	0.8450
		5	Sato instant rice	−0.0045	0.1189	−0.0375	0.9707
	II Wheat-based commodities	6	Yamazaki bread	0.5945	0.1490	3.9906	0.0018 **
		7	long-life bread	0.0367	0.0402	0.9142	0.3786
		8	cracker	0.0292	0.0225	1.3004	0.2179
		9	cake	0.0252	0.0233	1.0790	0.3018
	III Other, etc.	10	canned food	0.0662	0.0313	2.1114	0.0564 *
		11	curry	−0.0141	0.0512	−0.2751	0.7879
		12	side dish	0.0376	0.0329	1.1431	0.2753
		13	milk powder	0.0019	0.0019	1.0030	0.3357
		14	fruit	2.8515	2.7086	1.0528	0.3132

Note: multiple comparisons were made by Duncan’s test, with the * and ** symbols indicating the level of significance at < 0.05 and 0.01, respectively.

**Table 4:** Results of Alpha rice.

Variable	Coefficient	Std. Error	*t*-Statistic	Prob.
C	117.6720	228.1217	0.515830	0.6153
X	0.066177	0.031342	2.111412	0.0564
R-square	0.270874	Mean dependent var		562.3571
Adjusted R-squared	0.210113	S.D. dependent var		369.0127
S.E. of regression	327.9621	Akaike info criterion		14.5524
Log likelihood	−99.88666	Schwarz criterion		14.64653
*F*-statistic	4.458061	Hannan–Quinn criterion		14.54679
Prob (*F*-statistic)	0.056390	Durbin–Watson stat		2.351963

**Table 5:** Results of Yamazaki bread.

Variable	Coefficient	Std. Error	*t*-Statistic	Prob.
C	−1572.489	4075.014	−0.385886	0.7063
X	1.203655	0.559881	2.149840	0.0527
R-square	0.278057	Mean dependent var		6515.643
Adjusted R-squared	0.217895	S.D. dependent var		6624.509
S.E. of regression	5858.497	Akaike info criterion		20.32074
Log likelihood	−140.2452	Schwarz criterion		20.41203
*F*-statistic	4.621810	Hannan–Quinn criterion		20.31229
Prob (*F*-statistic)	0.052658	Durbin–Watson stat		1.144110

**Table 6:** Results of canned food.

Variable	Coefficient	Std. Error	*t*-Statistic	Prob.
C	−1766.724	1084.228	−1.629476	0.1292
X	0.594463	0.148966	3.990593	0.0018
R-square	0.570275	Mean dependent var		2227.857
Adjusted R-squared	0.534465	S.D. dependent var		2284.554
S.E. of regression	1558.755	Akaike info criterion		17.67273
Log likelihood	−121.7091	Schwarz criterion		17.76402
*F*-statistic	15.92483	Hannan–Quinn criterion		17.66428
Prob (*F*-statistic)	0.01792	Durbin–Watson stat		2.094655

## References

[1] Holguín-Veras J., Pérez N., Ukkusuri S., Wachtendorf T., Brown B. (2007). Emergency logistics issues affecting the response to katrina: A synthesis and preliminary suggestions for improvement. Transp. Res. Rec. J. Transp. Res. Board.

[2] Haghani A., Oh S.-C. (1996). Formulation and solution of a multi-commodity, multi-modal network flow model for disaster relief operations. Transp. Res. Part A Policy Pract..

[3] Ma J. Coronavirus: China Red Cross under Fire over Poor Distribution of Masks, Medical Supplies. https://www.scmp.com/news/china/society/article/3048512/china-red-cross-under-fire-poor-delivery-coronavirus-supplies.

[4] Shao J., Liang C., Wang X. (2020). Relief demand calculation in humanitarian logistics using material classification. Int. J. Environ. Res. Public Health.

[5] Tufekci S., Wallace W.A. (1998). The emerging area of emergency management and engineering. Ieee Trans. Eng. Manag..

[6] Rawls C.G., Turnquist M.A. (2010). Pre-positioning of emergency supplies for disaster response. Transp. Res. Part B.

[7] Holguín-Veras J., Jalle M. (2012). Immediate resource requirements after hurricane katrina. Nat. Hazards Rev..

[8] Picciano J. (2002). Responding to the Unexpected–Identifying Potential Technologies, Research and Development.

[9] Balcik B., Beamon B.M. (2008). Facility location in humanitarian relief. Int. J. Logist..

[10] Ozdamar L., Ekinci E., Kücükyazici B. (2004). Emergency logistics planning in natural disasters. Ann. Oper. Res..

[11] Rivera-Royeroa D., Galindob G., Yie-Pinedoa R. (2020). Planning the delivery of relief supplies upon the occurrence of a natural disaster while considering the assembly process of the relief kits. Socio-Econ. Plan. Sci..

[12] Zhao L., Li H., Sun Y. (2017). Planning emergency shelters for urban disaster resilience: An integrated location-allocation modeling approach. Sustainability.

[13] Kemball-Cook D., Stephenson R. (1984). Lessons in logistics from somalia. Disasters.

[14] Barbarosoglu G., Arda Y. (2004). A two-stage stochastic programming framework for transportation planning in disaster response. J. Oper. Res. Soc..

[15] Balcik B., Yanıkoğlu İ. (2020). A robust optimization approach for humanitarian needs assessment planning under travel time uncertainty. Eur. J. Oper. Res..

[16] Son C., Sasangohar F., Larsen E.P. (2019). Resilient performance of emergency department: Patterns, models and strategies. Saf. Sci..

[17] Rodríguez-Espíndola O., Albores P., Brewster C. (2018). Disaster preparedness in humanitarian logistics: A collaborative approach for resource management in floods. Eur. J. Oper. Res..

[18] Cavdur F., Sebatli A. (2019). A decision support tool for allocating temporary-disaster-response facilities. Decis. Support Syst..

[19] Kazama M., Noda T. (2012). Damage statistics (summary of the 2011 off the pacific coast of tohoku earthquake damage). Soils Found..

[20] Mori N., Takahashi T., Yasuda T., Yanagisawa H. (2011). Survey of 2011 tohoku earthquake tsunami inundation and run-up. Geophys. Res. Lett..

[21] Gideon M.R. Map Showing Major Areas of Damage in Japan, Including Fukushima & Missing Trains #Prayforjapan. https://100gf.wordpress.com/2011/03/13/map-showing-major-areas-of-damage-in-japan-including-fukushima-missing-trains-prayforjapan/.

[22] Saotome A., Numada M., Meguro K. (2012). Changes of contents and amount of relief goods during the 2011 off the Pacific coast of Tohoku earthquake--case study of the relief goods distributed in Sendai city. Jsce J. Earthq. Eng..

[23] Zhang X., Liu Y., Yang M. (2013). Comparative study of four time series methods in forecasting typhoid fever incidence in china. PLoS ONE.

[24] Ziegel E.R. (1992). Time series: Theory and methods (2nd ed,). Technometrics.

[25] Gujarati D.N. (2003). Basic Econometrics.

[26] Hahn H., Meyer-Nieberg S., Pickl S. (2009). Electric load forecasting methods: Tools for decision making. Eur. J. Oper. Res..

[27] Brandt S. (2014). The Method of Least Squares.

[28] Zhu X., Zhang G., Sun B. (2019). A comprehensive literature review of the demand forecasting methods of emergency resources from the perspective of artificial intelligence. Nat. Hazards.

[29] Xu X., Qi Y., Hua Z. (2010). Forecasting demand of commodities after natural disasters. Expert Syst. Appl..

[30] Sheu J.B. (2010). Dynamic relief-demand management for emergency logistics operations under large-scale disasters. Transp. Res. Part E: Logist. Transp. Rev..

